# Supplementary material for: Effect of minimally invasive autopsy and ethnic background on acceptance of clinical postmortem investigation in adults
Source: PLoS One. 2020 May 11;15(5):e0232944. doi: 10.1371/journal.pone.0232944 (PMC7213690; doi:10.1371/journal.pone.0232944)
Supplement: Attachment — Submitted filename: PONE-D-19-23443_reviewer.pdf [file pone.0232944.s001.pdf]

PLOS ONE

Effect of minimally invasive autopsy and ethnic background on consent rate for postmortem investigation in adult deceased patients: a prospective single center before-after study

--Manuscript Draft--

**Manuscript Number:**	PONE-D-19-23443
**Article Type:**	Research Article
**Full Title:**	Effect of minimally invasive autopsy and ethnic background on consent rate for postmortem investigation in adult deceased patients: a prospective single center before-after study
**Short Title:**	Effect of minimally invasive autopsy and ethnic background on postmortem consent rate
**Corresponding Author:**	Ivo M Wagensveld, M.D.<br>Erasmus MC<br>Rotterdam, Zuid Holland NETHERLANDS
**Keywords:**	autopsy; Virtual autopsy; Computed tomography; magnetic resonance imaging; autopsy consent rates
**Abstract:**	Objectives: Autopsy rates worldwide have dropped significantly over the last five decades. Imaging based autopsies are increasingly used as alternatives to conventional autopsy (CA). The aim of this study was to investigate the effect of the introduction of minimally invasive autopsy, consisting of CT, MRI and tissue biopsies on the total consent rate for postmortem diagnostics.<br><br>Methods: We performed a prospective single center before-after study. The intervention was the introduction of minimally invasive autopsy as an alternative to conventional autopsy. Minimally invasive autopsy consisted of MRI, CT, and CT-guided tissue biopsies. Consent rates over time and the effect of introducing minimally invasive autopsy were analyzed with a linear regression model. We performed a subgroup analysis comparing the consent rates of two groups: a group of western-European ethnicity versus a group of other ethnicities.<br><br>Results: Autopsy rates declined from 14.0% in 2010 to 8.3% in 2019. The linear regression model showed a significant effect of both time and availability of minimally invasive autopsy on the consent rate. The predicted acceptance rate in the model started at 15.1% in 2010 and dropped approximately 0.1% per month (β=-0.001, p< 0.001). Availability of minimally invasive autopsy increased the acceptance rate by 2.4% (β=0.024, p< 0.001). The overall post-mortem consent rate of people with an ethnic background other than western-European was significantly higher in years when minimally invasive autopsy was available compared to when it was not available (22/176=12.5% vs. 81/1014 (8.0%), p=0.049).<br><br>Conclusions: The introduction of minimally invasive autopsy had a small, but significant effect on the overall consent rates for postmortem investigations. Furthermore, the minimally invasive autopsy appears to be more acceptable than conventional autopsy among people with an ethnicity other than western-European.
**Order of Authors:**	Ivo M Wagensveld, M.D.
	Annick C Weustink
	Jan A Kors
	Britt M Blokker
	MG Myriam Hunink
	J Wolter Oosterhuis
**Additional Information:**	
**Question**	**Response**

Powered by Editorial Manager® and ProduXion Manager® from Aries Systems Corporation

**Financial Disclosure**<br><br>Enter a financial disclosure statement that describes the sources of funding for the work included in this submission. Review the submission guidelines for detailed requirements. View published research articles from *PLOS ONE* for specific examples.<br><br>This statement is required for submission and **will appear in the published article** if the submission is accepted. Please make sure it is accurate.<br><br>**Unfunded studies**<br>Enter: *The author(s) received no specific funding for this work.*<br><br>**Funded studies**<br>Enter a statement with the following details:<br>• Initials of the authors who received each award<br>• Grant numbers awarded to each author<br>• The full name of each funder<br>• URL of each funder website<br>• Did the sponsors or funders play any role in the study design, data collection and analysis, decision to publish, or preparation of the manuscript?<br>• **NO** - Include this sentence at the end of your statement: *The funders had no role in study design, data collection and analysis, decision to publish, or preparation of the manuscript.*<br>• **YES** - Specify the role(s) played.<br><br>* typeset	This work was supported by Erasmus Medical Centre Health Care Efficiency (grant 2010-10112), Erasmus MC Vriendenfonds (grant 104117), and Stichting Coolsingel (grant 255). The funders had no role in study design, data collection and analysis, decision to publish, or preparation of the manuscript.
**Competing Interests**<br><br>Use the instructions below to enter a competing interest statement for this submission. On behalf of all authors, disclose any competing interests that could be perceived to bias this work—acknowledging all financial support and any other relevant financial or non-financial competing interests.<br><br>This statement **will appear in the published article** if the submission is accepted. Please make sure it is	The authors have declared that no competing interests exist.

accurate. View published research articles from *PLOS ONE* for specific examples.<br><br>**NO authors have competing interests**<br><br>Enter: *The authors have declared that no competing interests exist.*<br><br>**Authors with competing interests**<br><br>Enter competing interest details beginning with this statement:<br><br>*I have read the journal's policy and the authors of this manuscript have the following competing interests: [insert competing interests here]*<br><br>* typeset	
**Ethics Statement**<br><br>Enter an ethics statement for this submission. This statement is required if the study involved:<br><br>• Human participants<br>• Human specimens or tissue<br>• Vertebrate animals or cephalopods<br>• Vertebrate embryos or tissues<br>• Field research<br><br>Write "N/A" if the submission does not require an ethics statement.<br><br>General guidance is provided below. Consult the submission guidelines for detailed instructions. **Make sure that all information entered here is included in the Methods section of the manuscript.**	This study was approved by the Erasmus MC Medical Ethical Committee (file number MEC-2011-055).

Powered by Editorial Manager® and ProduXion Manager® from Aries Systems Corporation

**Format for specific study types**<br><br>**Human Subject Research (involving human participants and/or tissue)**<br>• Give the name of the institutional review board or ethics committee that approved the study<br>• Include the approval number and/or a statement indicating approval of this research<br>• Indicate the form of consent obtained (written/oral) or the reason that consent was not obtained (e.g. the data were analyzed anonymously)<br><br>**Animal Research (involving vertebrate animals, embryos or tissues)**<br>• Provide the name of the Institutional Animal Care and Use Committee (IACUC) or other relevant ethics board that reviewed the study protocol, and indicate whether they approved this research or granted a formal waiver of ethical approval<br>• Include an approval number if one was obtained<br>• If the study involved *non-human primates*, add *additional details* about animal welfare and steps taken to ameliorate suffering<br>• If anesthesia, euthanasia, or any kind of animal sacrifice is part of the study, include briefly which substances and/or methods were applied<br><br>**Field Research**<br><br>Include the following details if this study involves the collection of plant, animal, or other materials from a natural setting:<br>• Field permit number<br>• Name of the institution or relevant body that granted permission	
**Data Availability**<br><br>Authors are required to make all data underlying the findings described fully available, without restriction, and from the time of publication. PLOS allows rare exceptions to address legal and ethical concerns. See the PLOS Data Policy and FAQ for detailed information.	No - some restrictions will apply

Powered by Editorial Manager® and ProduXion Manager® from Aries Systems Corporation

A Data Availability Statement describing where the data can be found is required at submission. Your answers to this question constitute the Data Availability Statement and **will be published in the article**, if accepted.<br><br>**Important:** Stating 'data available on request from the author' is not sufficient. If your data are only available upon request, select 'No' for the first question and explain your exceptional situation in the text box.<br><br>Do the authors confirm that all data underlying the findings described in their manuscript are fully available without restriction?	
**Describe where the data may be found in full sentences. If you are copying our sample text, replace any instances of XXX with the appropriate details.**<br><br>• If the data are **held or will be held in a public repository**, include URLs, accession numbers or DOIs. If this information will only be available after acceptance, indicate this by ticking the box below. For example: *All XXX files are available from the XXX database (accession number(s) XXX, XXX.).*<br>• If the data are all contained **within the manuscript and/or Supporting Information files**, enter the following: *All relevant data are within the manuscript and its Supporting Information files.*<br>• If neither of these applies but you are able to provide **details of access elsewhere**, with or without limitations, please do so. For example:<br><br>*Data cannot be shared publicly because of [XXX]. Data are available from the XXX Institutional Data Access / Ethics Committee (contact via XXX) for researchers who meet the criteria for access to confidential data.*<br><br>*The data underlying the results presented in the study are available from (include the name of the third party*	Some data cannot be shared publicly because of the confidential nature (i.e. surnames). These data are available upon request at the Erasmus Medical Center Clinical trial bureau of the radiology department (contact via imaging.trialbureau@erasmusmc.nl) for researchers who meet the criteria for access to confidential data.

and contact information or URL).<br>• This text is appropriate if the data are owned by a third party and authors do not have permission to share the data.<br><br>* typeset	
Additional data availability information:	

Manuscript Click here to access/download;Manuscript;Manuscript.docx

Effect of minimally invasive autopsy and ethnic background on consent rate for postmortem investigation in adult deceased patients: a prospective single center before-after study

IM Wagensveld[1,2], AC Weustink[1,2], JA Kors[3], BM Blokker[1,2], MGM Hunink[1,4,5], JW Oosterhuis[2]

[1] Department of Radiology and Nuclear Medicine, Erasmus University Medical Centre, PO Box 2040, 3000 CA Rotterdam, The Netherlands

[2] Department of Pathology, Erasmus University Medical Centre, PO Box 2040, 3000 CA Rotterdam, The Netherlands

[3] Department of Medical Informatics, Erasmus University Medical Centre, PO Box 2040, 3000 CA Rotterdam, The Netherlands

[4] Department of Epidemiology and Biostatistics, Erasmus University Medical Centre, PO Box 2040, 3000 CA Rotterdam, The Netherlands

[5] Centre for Health Decision Science, Harvard T.H. Chan School of Public Health, Harvard University, Boston, USA

*Corresponding author

Email: i.wagensveld@erasmusmc.nl

Abstract

Objectives

Autopsy rates worldwide have dropped significantly over the last five decades. Imaging based autopsies are increasingly used as alternatives to conventional autopsy (CA). The aim of this study was to investigate the effect of the introduction of minimally invasive autopsy, consisting of CT, MRI and tissue biopsies on the total consent rate for postmortem diagnostics.

Methods

We performed a prospective single center before-after study. The intervention was the introduction of minimally invasive autopsy as an alternative to conventional autopsy. Minimally invasive autopsy consisted of MRI, CT, and CT-guided tissue biopsies. Consent rates over time and the effect of introducing minimally invasive autopsy were analyzed with a linear regression model. We performed a subgroup analysis comparing the consent rates of two groups: a group of western-European ethnicity versus a group of other ethnicities.

Results

Autopsy rates declined from 14.0% in 2010 to 8.3% in 2019. The linear regression model showed a significant effect of both time and availability of minimally invasive autopsy on the consent rate. The predicted acceptance rate in the model started at 15.1% in 2010 and dropped approximately 0.1% per month ($\beta = -0.001$, $p < 0.001$). Availability of minimally invasive autopsy increased the acceptance rate by 2.4% ($\beta = 0.024$, $p < 0.001$). The overall post-mortem consent rate of people with an ethnic background other than western-European was significantly higher in years when minimally invasive autopsy was available compared to when it was not available (22/176=12.5% vs. 81/1014 (8.0%), p=0.049).

Conclusions

The introduction of minimally invasive autopsy had a small, but significant effect on the overall consent rates for postmortem investigations. Furthermore, the minimally invasive autopsy appears to be more acceptable than conventional autopsy among people with an ethnicity other than western-European.

Introduction

Clinical autopsy acceptance rates have dropped considerably over the previous decades. (1-6) Improvements in imaging techniques in living patients may have led to the belief that autopsies hardly add to the information acquired prior to death. (7) However, despite improved diagnostic studies, autopsies still provide valuable feedback on diagnoses and treatment, and accurate statistics on causes of death. (8, 9) Moreover, they are useful for healthcare policymaking, education and research purposes. (3, 10-12)

The low consent rate of next-of-kin for the autopsy is one of the reasons for the decline in clinical autopsy rates. Therefore, strategies to improve the consent rates are under investigation: improved availability of modern imaging techniques has led to the development of imaging-based autopsy techniques. Such methods can be non-invasive or minimally invasive (9); in our hospital we introduced and validated a minimally invasive autopsy, consisting of postmortem CT, MRI and CT-guided biopsies. (8)

The aim of our study was to determine the acceptance rates of postmortem investigations (both minimally invasive and conventional autopsy combined) on adult deceased patients in our hospital over the years 2010-2019 and to investigate if the introduction of the minimally invasive autopsy led to an increase in acceptance of postmortem investigations. A subgroup analysis was performed comparing a group of western-European ethnicity with a group of other ethnicities. Furthermore, we used a questionnaire to investigate the considerations and motivations of doctors and next-of-kin in the consent process for postmortem investigations.

Materials and Methods

Setting and design

This study was performed at the Erasmus University Medical Center in Rotterdam, the largest academic hospital in the Netherlands. The design was a prospective before-after study whereby additional data was collected retrospectively. The intervention was the introduction of minimally invasive autopsy as an alternative to conventional autopsy. The study was approved by the Erasmus University Medical Center Medical Ethical Committee (file number MEC-2011-055). The institutional review board approved the study prior to data collection. All adult patients who had died in-hospital were included.

Autopsy procedures

Conventional autopsy and minimally invasive autopsy were both available from Monday to Friday. Only 1 minimally invasive autopsy per day was possible, due to limited scanner availability. When multiple minimally invasive autopsy procedures were requested on the same day, requests would be processed in the order they were received: if next-of-kin agreed, a procedure would be postponed until the next available day.

Minimally invasive autopsy procedure

A minimally invasive autopsy consisted of MRI of the head and torso, full-body CT scan and CT-guided biopsies of organs (heart, lungs, liver, kidneys and spleen) and additional biopsies of abnormal / pathological lesions detected on imaging. The CT and MRI protocols are described in previous articles. (8, 13-15)

Conventional autopsy procedure

The conventional autopsy was performed according to standard department protocol: the body was opened with a Y-incision and the thoracic cavity opened with a rib-cutter. Organs were eviscerated by the mortuary assistant and dissected by a resident in pathology, supervised by a certified pathologist. (8)

Acceptance

The primary outcome measure was the effect of minimally invasive autopsy on the consent rate for postmortem diagnostics.

Consent for both autopsy procedures was requested by the treating physicians. Before the actual introduction of the minimally invasive autopsy, all clinical wards were educated about the new autopsy method.

Questionnaires

As a secondary outcome we investigated the motivations of next-of-kin for consenting to or refusing an autopsy, and the motivations of doctors to not ask for permission. We distributed the questionnaires to the doctors who were involved in the consent process, since they would be informed about the motivations of next-of-kin when discussing the possibility of autopsy after a patient had passed away. Questionnaires were distributed from September 2016 - December 2017.

Data analysis

Because we expected that the autopsy rates were declining during the study period we performed a linear regression analysis to calculate the effect of time and availability of minimally invasive autopsy on consent rate. The independent variables were time in months since the start of the study and the availability of minimally invasive autopsy as a standalone postmortem investigation.

We performed a subgroup analysis among people of western-European ethnicity versus people of other ethnical backgrounds. We calculated the acceptance rates of both groups and performed an independent T-test in order to test for significance.

To determine the ethnicity, we used a two-stage classification process. In the first stage, the predicted probabilities of a supervised machine-learning algorithm were used to distinguish between classifications with high and low certainty. For this stage, we used a random forest classifier (method ranger in the R package caret). To train and test the classifier, we used data from a questionnaire in which the next-of-kin were asked to provide the ethnic background of the deceased. (7, 16) The total set consisted of 2,083 cases, which were split in a training set (80%) and a test set (20%). As features we used character n-grams (with n = 2, 3, and 4) of the last names and, if available, of the first names. Each case was labelled as having either a western-European or other ethnic background. Henceforth we will refer to this latter group as 'other' ethnicities. The training set was used to develop the classifier; the test set was only used for performance evaluation. Performance measures were the area under the receiver operating characteristic curve (AUROC), negative predictive value (proportion of correctly predicted Western-European cases), and positive predictive value (proportion of correctly predicted cases of 'other' ethnicity). (17-19) In the second stage, the classifications with low certainty were manually validated, in cases of doubt the Dutch surname database was consulted (https://www.cbgfamilienamen.nl/nfb/). For the manual classification, the group allocation (intervention vs. non-intervention) was unknown to the observer (IMW).

Results

Consent rates

Autopsy rates declined from 14.0% in 2010 to 8.3% in 2019. The annual autopsy rates of the years 2010-2019 are shown in Figure 1.

In the 'intervention' period (when minimally invasive autopsy was available) from October 2016 through December 2017, 1056 adult patients died and permission for postmortem diagnostics was given in 133 cases (12.6%): 87 underwent conventional autopsy (8.2%) and 46 minimally invasive autopsy (4.4%).

The linear regression model showed a significant effect on the consent rate of both time and availability of minimally invasive autopsy. The predicted acceptance rate in the model started at 15.1% in September 2010 and dropped approximately 0.1% per month ($\beta = -0.001$, $p < 0.001$) and minimally invasive autopsy availability increased the acceptance rate by 2.4% ($\beta = 0.024$, $p < 0.001$).

Fig 1. Postmortem consent rates 2010-2019

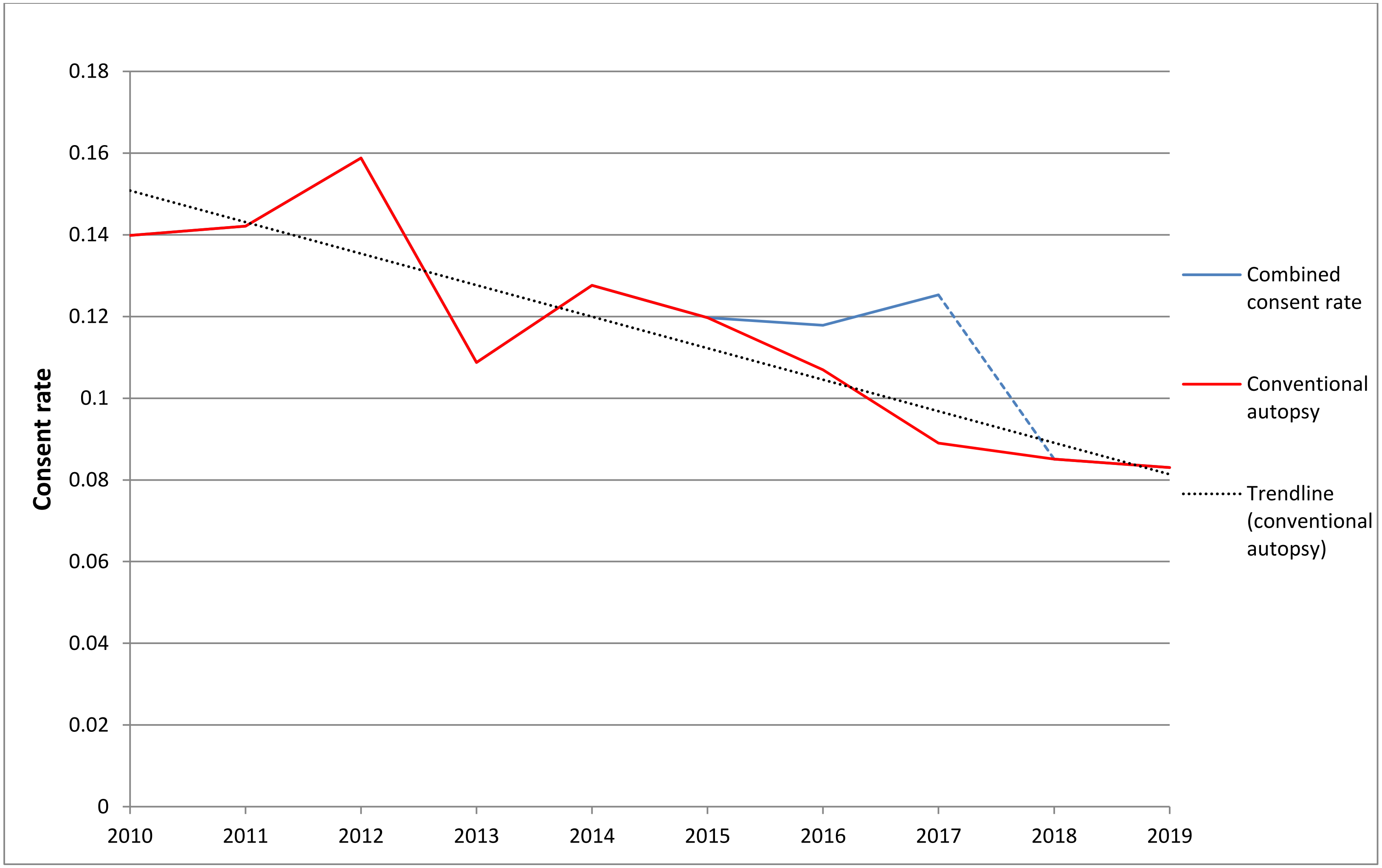

Legend to Fig 1. Combined consent rate are the combined consent rates of minimally invasive autopsy and conventional autopsy

Prediction of ethnicity

We used 5-fold cross-validation to train the random forest classifier on the training set. On the test set, this classifier obtained an area under the receiver operating characteristic curve of 0.91, showing good performance. We empirically set probability thresholds to distinguish between high and low confidence classifications. Names with a predicted probability lower than 0.50 were labeled as ’other’ ethnicity (this threshold yielded a positive predictive value of 1 on the test set), and names with a predicted probability greater than 0.82 were labeled as Western-European (yielding a negative predictive value of 0.95 on the test set). Cases with a probability between 0.50 and 0.82 were manually validated, using the Dutch surnames database. Of the cases from 2010-2019 which were not part of the learning dataset (n=4764), 1548 cases had a probability between 0.5 and 0.82; 733 (47.4%) of these were manually scored as western-European. In the total cohort, 82.8% (5736/6928) were classified as western-European.

Effect of ethnic background on consent rate

The consent rates of western-Europeans and ‘other’ ethnicities in the different cohorts is detailed in table 1. In the years when minimally invasive autopsy was not available, western-Europeans had a significantly higher consent rate for conventional autopsy than ‘other’ ethnicities (629/4858=12.9% vs. 82/1014=8.0%, p<0.001). When minimally invasive autopsy was available the total consent rate for postmortem investigations was nearly the same for western-Europeans and ‘other’ ethnicities (111/880=12.6% vs. 22/176=12.5%, p=0.97). The post-mortem consent rate among ’other’ ethnicities was significantly higher in the years when minimally invasive autopsy was available compared to the years when it was not available (22/176=12.5% vs. 81/1014=8.0%, p=0.049).

Table 1. Consent rates of western-European vs 'other' ethnicities.

Time period	**Available procedures**	**n**		**western-European**	**'other' ethnicities**	**Overall consent rate**	**Western-European vs 'other' ethnicities**
Pre-intervention (Jan. 2010 - Sept. 2016)	CA only	4679	CA consent rate	538/3875 (13.9%)	71/804 (8.8%)	609/4679 (13.0%)	p>0.001
Post-intervention (Jan. 2018- Mar. 2019)	CA only	1193	CA consent rate	90/982 (9.2%)	11/211 (5.2%)	101/1193 (8.5%)	p=0.061
Total non-intervention (2010-2019, excluding intervention period)	CA only	5872	CA consent rate	**629/4858 (12.9%)**	**81/1014 (8.0%)**	**710/5872 (12.1%)**	p>0.001
Intervention (Oct. 2016 - Dec. 2017)	MIA + CA	1056	MIA consent rate	37/880 (4.2%)	9/176 (5.1%)	46/1056 (4.4%)	p=0.59
	MIA + CA	1056	CA consent rate	74/880 (8.4%)	13/176 (7.4%)	87/1056 (8.2%)	p=0.45
	MIA + CA	1056	Total consent rate (MIA + CA)	**111/880 (12.6%)**	**22/176 (12.5%)**	**133/1056 (12.6%)**	p=0.97
Comparison of total consent rate: intervention vs. non intervention				p=0.79	p=0.049	p=0.65	

Questionnaires

Questionnaires were distributed from September 2016 to December 2017. 505 out of 1123 (45.9%) questionnaires were returned. In the group that refused conventional autopsy or minimally invasive autopsy 413/988 (41.8%) questionnaires were returned, and in the group that gave permission 92/135 (68.1%) questionnaires were returned. In the group that gave permission for minimally invasive autopsy 40/46 (87.0%) of questionnaires were returned and in the group that gave permission for conventional autopsy 55/92 (59.8%) questionnaires were returned.

In 436/505 (86.3%) cases the doctors involved in the consent process declared that they had requested permission for autopsy. Doctors' reasons not to ask for permission are listed in table 2, the most frequently heard reason was "the cause of death is already known" 32/69 (46.4%).

Table 2: Reasons why doctors did not ask for permission

Motivation	Frequency
The cause of death is already known	32/69 (46.4%)
The next-of-kin had already consented to an organ donation procedure	9/69 (13%)
Perceived uncomfortable situation	7/69 (10%)
No family present to ask permission	6/69 (9%)
Doctor thought an autopsy would be too much to ask	6/69 (9%)

Reasons of next-of-kin for giving or denying consent for postmortem diagnostics are listed in table 3 and 4.

Table 3: Reasons of next-of-kin for giving consent

Motivation	Frequency
To find out the cause of death	65/92 (70.7%)
Wanting to know the severity of disease	17/92 (18.5%)
Cater to treating doctor's request	16/92 (17.4%)
Testing for hereditary disorders	13/92 (14.1%)
Testing for presence of diseases, not related to the cause of death	10/92 (10.7%)
Contribute to scientific research and/or medical knowledge	7/92 (7.6%)
Other reasons	4/92 (4.3%)

Table 4: Reasons of next-of-kin for denying consent*

Motivation	Frequency
The cause of death is already known	166/339 (49.0%)
Long illness, "the deceased has suffered enough"	77/339 (22.7%)
Religious motivation	35/339 (10.3%)
Autopsy is considered too invasive, scary or macabre	35/339 (10.3%)
Autopsy would take too long	8/339 (2.4%)
Already consented to donation procedure	7/339 (2.1%)
No reason given	24/339 (7.1%)

* this category also contains answers from next-of-kin who gave consent for one of the autopsy methods, but nevertheless gave objections against postmortem diagnostics.

The reasons for consenting to an autopsy procedure mostly overlapped for both conventional autopsy and minimally invasive autopsy. One exception was that people consenting to minimally invasive autopsy did so more often “to contribute to research and/or contribute to medical knowledge” than those consenting to conventional autopsy (5/46=10.9% vs. 2/99=2.0%, p=0.02).

Discussion

In this study we investigated if the introduction of a minimally invasive autopsy, consisting of CT, MRI and tissue biopsies, would increase the total consent rate for postmortem diagnostics. We found that the introduction of minimally invasive autopsy had a small, but significant effect on the combined consent rates (conventional autopsy and minimally invasive autopsy) for postmortem investigations. This is important information in light of the decline in autopsy consent rates, which is observed all over the world since approximately 1950. In our hospital we saw a further decrease in consent rates of approximately 0.1% per month since the start of our study. (20)

The measured effect (2.4%) that minimally invasive autopsy had on consent rates was smaller than we had expected. The reason for this is unclear: perhaps the invasiveness of the procedure isn’t as important a motivation to deny consent for the next-of-kin. Alternatively, minimally invasive autopsy, using biopsies, might still be considered too invasive; or maybe any additional procedures performed on the deceased’s body are considered macabre or scary. Another reason might be that low autopsy rates are attributable to a low request rate (21). In our hospital request rates are reported to be very high, but these rates are self-reported by doctors and the real request rate might be lower than what is reported.

An important reason we introduced the minimally invasive autopsy in our hospital is that the population of Rotterdam, where this study was carried out, consists for a large part of non-western immigrants (around 38% in 2018). Although most religions do not outrightly prohibit autopsy, in many religious groups some inhibitions against postmortem investigations are present. (22-24) In the years when minimally invasive autopsy was not available as a stand-alone postmortem investigation we observed that the consent rate in people of ethnicities other than western-European was significantly lower than in people with a western-European ethnicity. When minimally invasive autopsy was available, the combined consent rates of minimally invasive autopsy and conventional autopsy were the same for non-western-Europeans and ‘other’ ethnicities (12.6% and 12.5% respectively), and the consent rate among ‘other’ ethnicities was significantly higher than in the years without minimally invasive autopsy (12.5%

vs 8.0%, p=0.049). This strongly suggests that minimally invasive autopsy is indeed more acceptable to people with an ethnicity other than western-European.

The main motivation for next-of-kin to withhold consent for autopsy was the assumption that the cause of death was already known, and no important questions remained to be answered. This is in line with our earlier questionnaire study. (7) It should be noted here that autopsy results differ from the presumed cause of death before autopsy in a substantial percentage of cases. Interestingly the invasiveness of the procedure was not often mentioned as motivation against postmortem diagnostics. This might suggest that the invasiveness is not a big factor in the declining autopsy rates, contrary to what is often believed. This could be part of the reason why the postmortem consent rate of western-Europeans was not higher when minimally invasive autopsy was available.

In a study by Cox et al. the authors achieved a substantial increase in autopsy rates (from 5% to 38%). They attributed this increase in autopsy rates mainly to the study setting, which resulted in improved logistics of the consent process and a big increase in the request rate of doctors. Both factors did not play a big role in our study. We did not make major changes to the logistics of the consent process and the logistics of conventional autopsy stayed the same. The autopsy request rate in our hospital was already reported to be high before the introduction of minimally invasive autopsy. Furthermore, in some cases of the study by Cox et al. a member of the research team asked for consent. (25) It has also been suggested that pathologists should personally ask next-of-kin for permission, because they are most informed about the different procedures and can most adequately answer any questions the next-of-kin might have. (26) In our study, however, the treating physician always requested consent. We educated the doctors about the method, prior to introducing the minimally invasive autopsy hospital wide. For further questions during the study period, a researcher would be available by phone at any time. Apart from that, there was limited direct involvement of the research team in the day-to-day consent process. We think that success like that of Cox et al. and a similar increase in consent rates requires a dedicated team in each hospital with in-depth knowledge of all available postmortem diagnostic methods and all

involved logistics. Members of this team could assist doctors in the consent process, or even request consent from next-of-kin in person, preferably with the treating physician present.

In studies performed in Sweden attitudes towards autopsy, organ donation and dissection (donation of the body for scientific purposes) were evaluated. Interestingly the authors found that at the time of the interview Swedes were much more positive about autopsy than they were about organ donation. (27, 28) This is in stark contrast with the current situation in the Netherlands and other western countries, where the autopsy rates are often below 10%, while registered organ donation is much more common (42% in 2018 in the Netherlands). (29) In new legislation, which will be implemented in 2020, all adults in the Netherlands are registered as an organ donor unless consent is specifically denied.

Another factor that can influence autopsy rates is the quality of the autopsy perceived by the doctors who request autopsy. It is necessary to facilitate close collaboration between clinicians and the autopsy team. This includes ensuring good communication beforehand about what can be expected of the autopsy and clear and timely information about the autopsy results afterwards. Furthermore, there must be clarity about the financial aspects of autopsies: clinicians should not have to fear that a high autopsy rate will lead to a high fee for their department. In general, an increase in autopsy rates will only be achieved if there is a positive attitude towards the autopsy in clinicians, pathologists and other involved parties. (30-32)

Limitations

A limitation of our study was a relatively low response rate to the questionnaires. In our experience making the questionnaires obligatory for doctors to fill in after death makes the quality of the responses worse. By making it optional the doctors and families with a more positive attitude towards autopsy are more inclined to respond to the questionnaire which may lead to a bias in the answers. Another limitation was that we asked the treating physician about the motivation of the next-of-kin, because we

considered it unethical to ask the bereaved family directly after their loss. This way the motivation of the next-of-kin was investigated indirectly, through the treating clinician.

In this study all ethnicities which were not classified as western European are classified in the category “other” ethnicities. The distinction between western-European ethnicity and all ‘other’ ethnicities is very broad and possibly semantically confusing. The western-European group does not include people from southern-, northern- and eastern-Europe and the USA, which are also considered as “western” in the common use of the word. Unfortunately, the group of other ethnicities was relatively small (less than 20% of the total cohort), therefore further subdividing that group resulted in low receiver operating characteristic curves for the supervised machine-learning algorithm. The Dutch surname database, used for the manual validation, contains the frequency of occurrence of surnames in 1947 and 2007. Most cases with an ‘other’ ethnicity were migrant workers who moved to the Netherlands after the second world war. This corresponds with the data from the Personal Records Database from the municipality of Rotterdam: over 50% of the population has a migration background, and within this group roughly 75% has a non-western background.

Information about the minimally invasive autopsy was distributed at the start of the cohort. Doctors in our hospital were already familiar with the procedure, because it had already been validated in the years prior to this cohort. Nevertheless, in a hospital environment with constant changes in personnel, the familiarity with minimally invasive autopsy and the ins-and-outs of this new procedure were suboptimal. We feel that, for a reliable measurement of the effect of minimally invasive autopsy on the total autopsy rates, a longer period of inclusion is necessary, so that doctors and the public become more familiar with the procedure. In this light, the results from our current cohort should be seen as a baseline measurement for our hospital and comparable hospitals.

Conclusion

In this study we investigated if the introduction of a minimally invasive autopsy consisting of CT, MRI and biopsies would lead to an increase in total combined consent rates for post-mortem investigations (conventional autopsy and minimally invasive autopsy). We found that the introduction of minimally invasive autopsy had a small, but significant effect on the combined consent rates for postmortem investigations. Furthermore, the minimally invasive autopsy appears to be more acceptable than conventional autopsy among people with an ethnicity other than western-European.

References

1. Shojania KG, Burton EC. The Vanishing Nonforensic Autopsy. New England Journal of Medicine. 2008;358(9):873-5.
2. Turnbull A, Osborn M, Nicholas N. Hospital autopsy: Endangered or extinct? J Clin Pathol. 2015;68(8):601-4.
3. Burton JL, Underwood J. Clinical, educational, and epidemiological value of autopsy. Lancet. 2007;369(9571):1471-80.
4. Blokker BM, Weustink AC, Hunink MGM, Oosterhuis JW. Autopsy rates in the Netherlands: 35 years of decline. PLOS ONE. 2017;12(6):e0178200.
5. Roberts WC. The autopsy: its decline and a suggestion for its revival. N Engl J Med. 1978;299(7):332-8.
6. Ayoub T, Chow J. The conventional autopsy in modern medicine. J R Soc Med. 2008;101(4):177-81.
7. Blokker BM, Weustink AC, Hunink MGM, Oosterhuis JW. Autopsy of Adult Patients Deceased in an Academic Hospital: Considerations of Doctors and Next-of-Kin in the Consent Process. PLOS ONE. 2016;11(10):e0163811.
8. Blokker BM, Weustink AC, Wagensveld IM, von der Thüsen JH, Pezzato A, Dammers R, et al. Conventional Autopsy versus Minimally Invasive Autopsy with Postmortem MRI, CT, and CT-guided Biopsy: Comparison of Diagnostic Performance. Radiology. 2018.
9. Blokker BM, Wagensveld IM, Weustink AC, Oosterhuis JW, Hunink MG. Non-invasive or minimally invasive autopsy compared to conventional autopsy of suspected natural deaths in adults: a systematic review. Eur Radiol. 2015.
10. Shojania KG, Burton EC, McDonald KM, Goldman L. Changes in rates of autopsy-detected diagnostic errors over time: a systematic review. JAMA. 2003;289(21):2849-56.
11. Reichert CM, Kelly VL. Prognosis for the autopsy. Health Aff (Millwood). 1985;4(2):82-92.
12. Turnbull A, Martin J, Osborn M. The death of autopsy? Lancet. 2015;386(10009):2141.
13. Wagensveld IM, Blokker BM, Wielopolski PA, Renken NS, Krestin GP, Hunink MG, et al. Total-body CT and MR features of postmortem change in in-hospital deaths. PLOS ONE. 2017;12(9):e0185115.
14. Wagensveld IM, Blokker BM, Pezzato A, Wielopolski PA, Renken NS, von der Thüsen JH, et al. Diagnostic accuracy of postmortem computed tomography, magnetic resonance imaging, and computed tomography-guided biopsies for the detection of ischaemic heart disease in a hospital setting. European Heart Journal - Cardiovascular Imaging. 2018;19(7):739-48.
15. Wagensveld IM, Hunink MGM, Wielopolski PA, van Kemenade FJ, Krestin GP, Blokker BM, et al. Hospital implementation of minimally invasive autopsy: A prospective cohort study of clinical performance and costs. PLOS ONE. 2019;14(7):e0219291.
16. Blokker BM, Weustink AC, Wagensveld IM, von der Thüsen JH, Pezzato A, Dammers R, et al. Conventional Autopsy versus Minimally Invasive Autopsy with Postmortem MRI, CT, and CT-guided Biopsy: Comparison of Diagnostic Performance. Radiology. 2018;289(3):658-67.
17. Mateos P. A review of name-based ethnicity classification methods and their potential in population studies. Population, Space and Place. 2007;13(4):243-63.
18. Ambekar A, Ward C, Mohammed J, Male S, Skiena S. Name-ethnicity classification from open sources. Proceedings of the 15th ACM SIGKDD international conference on Knowledge discovery and data mining; Paris, France. 1557032: ACM; 2009. p. 49-58.
19. Treeratpituk P, Giles CL. Name-ethnicity classification and ethnicity-sensitive name matching. Proceedings of the Twenty-Sixth AAAI Conference on Artificial Intelligence; Toronto, Ontario, Canada. 2900890: AAAI Press; 2012. p. 1141-7.
20. Crowley PF, McKelvie PA. The decline in hospital autopsy rates. Med J Aust. 1996;164(3):188-9.
21. Sherwood SJ, Start RD. Asking relatives for permission for a post mortem examination. Postgraduate medical journal. 1995;71(835):269-72.

22. Gatrad AR. Muslim customs surrounding death, bereavement, postmortem examinations, and organ transplants. BMJ. 1994;309(6953):521.
23. Lewis C, Latif Z, Hill M, Riddington M, Lakhanpaul M, Arthurs OJ, et al. "We might get a lot more families who will agree": Muslim and Jewish perspectives on less invasive perinatal and paediatric autopsy. PLOS ONE. 2018;13(8):e0202023.
24. Lewis C, Hill M, Arthurs OJ, Hutchinson JC, Chitty LS, Sebire N. Health professionals' and coroners' views on less invasive perinatal and paediatric autopsy: a qualitative study. Archives of Disease in Childhood. 2018;103(6):572.
25. Cox JA, Lukande RL, Kateregga A, Mayanja-Kizza H, Manabe YC, Colebunders R. Autopsy acceptance rate and reasons for decline in Mulago Hospital, Kampala, Uganda. Trop Med Int Health. 2011;16(8):1015-8.
26. McDermott MB. Obtaining consent for autopsy. BMJ (Clinical research ed). 2003;327(7418):804-6.
27. Mjornheim B, Rosendahl A, Eriksson LC, Takman C. Attitudes of Nurses and Physicians About Clinical Autopsy in Neonatal and Adult Hospital Care: A Survey in Sweden. Nurs Res. 2015;64(4):264-71.
28. Sanner M. A Comparison of Public Attitudes Toward Autopsy, Organ Donation, and Anatomic Dissection: A Swedish Survey. JAMA. 1994;271(4):284-8.
29. https://www.cbs.nl/en-gb/news/2018/32/donor-register-includes-6-3-million-persons.
30. Haque AK, Patterson RC, Grafe MR. High autopsy rates at a university medical center. What has gone right? Arch Pathol Lab Med. 1996;120(8):727-32.
31. Haque AK. The autopsy and the public need. Arch Pathol Lab Med. 1995;119(12):1092-4.
32. Langer R, Tröhler A, Schnüriger B, Trippel M, Blank A, Banz Y, et al. Implementation of modern tools in autopsy practice—the way towards contemporary postmortal diagnostics2018.
