# Supplementary material for: Effect of minimally invasive autopsy and ethnic background on acceptance of clinical postmortem investigation in adults
Source: PLoS One. 2020 May 11;15(5):e0232944. doi: 10.1371/journal.pone.0232944 (PMC7213690; doi:10.1371/journal.pone.0232944)
Supplement: Attachment — Submitted filename: Response to reviewers.docx [file pone.0232944.s002.docx]

Dear sir / madam,

Firstly, on behalf of all the authors, I would like to thank the editor and the reviewers for their valuable comments. Answers to the questions of the Plos One staff and the reviewers can be found below.

1. Please ensure that your manuscript meets PLOS ONE's style requirements, including those for file naming. The PLOS ONE style templates can be found at

<http://www.journals.plos.org/plosone/s/file?id=wjVg/PLOSOne_formatting_sample_main_body.pdf> and <http://www.journals.plos.org/plosone/s/file?id=ba62/PLOSOne_formatting_sample_title_authors_affiliations.pdf>

2. Please provide additional details regarding participant consent.

In the ethics statement in the Methods and online submission information, please ensure that you have specified (a) whether consent was informed and (b) what type you obtained (for instance, written or verbal, and if verbal, how it was documented and witnessed).

If your study included minors, state whether you obtained consent from parents or guardians.

If the need for consent was waived by the ethics committee, please include this information.

Answer:
We added a paragraph in the methods section about the consent procedure. For all cases that underwent postmortem investigation written informed consent was obtained from the next-of-kin. Cases for which no permission was obtained were not ‘included’ in our study. For the latter only anonymized, generally accessible data from hospital-wide statistics were used.

3. We note that you have indicated that data from this study are available upon request. PLOS only allows data to be available upon request if there are legal or ethical restrictions on sharing data publicly. For information on unacceptable data access restrictions, please see <http://journals.plos.org/plosone/s/data-availability#loc-unacceptable-data-access-restrictions>.

In your revised cover letter, please address the following prompts:

a) If there are ethical or legal restrictions on sharing a de-identified data set, please explain them in detail (e.g., data contain potentially identifying or sensitive patient information) and who has imposed them (e.g., an ethics committee). Please also provide contact information for a data access committee, ethics committee, or other institutional body to which data requests may be sent.

Answer:
Data requests can be sent to the radiology trial agency of the Erasmus Medical Centre at: imaging.trialbureau@erasmusmc.nl

b) If there are no restrictions, please upload the minimal anonymized data set necessary to replicate your study findings as either Supporting Information files or to a stable, public repository and provide us with the relevant URLs, DOIs, or accession numbers. Please see <http://www.bmj.com/content/340/bmj.c181.long> for guidelines on how to de-identify and prepare clinical data for publication. For a list of acceptable repositories, please see <http://journals.plos.org/plosone/s/data-availability#loc-recommended-repositories>.

We will update your Data Availability statement on your behalf to reflect the information you provide.

Answer:
Our results are based on generally accessible hospital wide statistics (i.e. consent rate for postmortem investigation), the anonymized dataset for this part would not contain more relevant data than already provided in the manuscript (e.g. a list of case numbers with consent or no consent). The list of surnames of all deceased patients that was used to predict ethnicity is obviously highly privacy sensitive information, therefore these data cannot be made publicly available.

Answers to reviewer questions:
Reviewer #1:
1. I think there is a need to revise the title. Currently, the title is little long.

Answer:
We changed the title to: ‘Effect of minimally invasive autopsy and ethnic background on acceptance of clinical postmortem investigation in adults’

2. In objectives (abstract section), you have not highlighted that you are also measuring the consent rates among different ethnicity. You might want to add that..

Answer:
Added this to the objectives section in the abstract.

3. In introduction section line no. 56, you have used the phrase" determining acceptance rates'; you can think of using this in your title. Whatever you choose to write; you should remain consistent.

Answer:
We use the word ‘acceptance’ in the new title. Where possible we changed the wording in the text to ‘acceptance’ instead of consent rate, however, the word acceptance has a broader meaning than just consent, therefore we use the word ‘consent’ for the situation of giving specific consent for the procedure and the word ‘acceptance’ for the values and opinions of next-of-kin with regards to postmortem diagnostics that lead to consenting to postmortem investigation.

Since in our study postmortem investigation was always carried out when consent was given, the terms ‘consent rate’ and ‘autopsy rate’ are interchangeable in terms of numbers. Therefore, we now consistently use the term ‘autopsy rate’ instead of ‘consent rate’ (where appropriate), in our methods, results and discussion section, because this term is most commonly used in the literature. Whenever we use the term ‘overall autopsy rate’ it includes conventional- and minimally invasive autopsy.

4. In introduction section; line no 60-62; you have added one more objective. This is not stated in your title and abstract. You might want to add it there too

Answer:
We added the objective to measure consent rate among different ethnicities in the objectives in the abstract. The questionnaires were only carried out as a secondary analysis and therefore we did not add those results to the abstract, to limit the amount of words in the abstract and to focus on the main objectives and results.

5.I think the description of MIA and conventional autopsy can be moved to introduction section from the methods section.

Result:
We feel that the description of the conventional autopsy and minimally invasive autopsy belongs in the methods section. A very concise description of the minimally invasive autopsy is already present in the introduction section.

6.I think in methods section, you should have the heading of primary and secondary outcomes; instead of acceptance and questionnaires

Answer:
We changed the headings in the methods section according to the suggestions of the reviewer.

7. Also, I think you should talk about the procedures in detail. How this was done. Method section needs more clarity.

Answer:
We made the choice to keep the procedure details about the different methods of the conventional autopsy and minimally invasive autopsy concise, because these are not relevant to the current study. In response to the reviewer, we added a line in the methods section that explains the main difference between the minimally invasive autopsy and the conventional autopsy. Any additional information can be found in the quoted previously published articles (among them publications in Plos One) if the reader is interested, but these details are not necessary for understanding our study or replicating the results.

8. I believe the discussion section needs some more work; in terms of comparing and contrasting similar studies that have been conducted on this domain.

Anwer: To the best of our knowledge this is the only study in which acceptance of MIA and CA was prospectively investigated. The outcome is consistent with the results of a questionnaire-study by Rutty et al. (J Forensic Legal Med 2011), which addressed the acceptance of post-mortem CT-scanning compared to invasive autopsy. Although only partially relevant for our study, it nonetheless demonstrates that the public overwhelmingly preferred CT-scanning over the conventional autopsy as method for post-mortem investigation in accordance with intuition. We added a paragraph at the start of the discussion about the comparison with the available literature.

9. It would be good if you can highlight some policy implications for this study.

Answer: There is consensus that autopsies are important for reliable health statistics for health care policymaking. In populations with a substantial proportion having a non-Western background the data will be biased towards the Western population to the disadvantage of the non-Western part. A way of addressing this problem is to apply autopsy techniques that are acceptable to both groups. Our study shows that with the MIA applied here the autopsy rates among Western and non-Western people are similar. We recommend that MIA should be offered in populations with a high proportion having a non-Western background, and in countries where the autopsy meets a great deal of objection, like in Islamic countries. We also recommend that MIA is carried out in specialized centers because of the expertise it requires on the part of radiologists and pathologists to take and interpret the image-guided biopsies, and the costliness of equipment (CT and MRI). We added a paragraph at the end of our discussion section with policy implications.

10. I have not commented on the results section and stats

Reviewer #2: It is a well written manuscript with sound statistical analysis. Just one suggestion to use word "Acceptance" in title of the paper

Answer:
We changed the title to:
‘Effect of minimally invasive autopsy and ethnic background on acceptance of clinical postmortem investigation in adults’
